# Facial Expressions of Threat Influence Perceived Gaze Direction in 8 Year-Olds

**DOI:** 10.1371/journal.pone.0049317

**Published:** 2012-11-14

**Authors:** Gillian Rhodes, Brooke Addison, Linda Jeffery, Michael Ewbank, Andrew J. Calder

**Affiliations:** 1 ARC Centre of Excellence in Cognition and its Disorders, School of Psychology, University of Western Australia, Crawley, Western Australia, Australia; 2 MRC Cognition and Brain Sciences Unit, Cambridge, United Kingdom; McMaster University, Canada

## Abstract

Adults show reciprocal influences between the perception of gaze direction and emotional expression. These facilitate the understanding of facial signals, because the meaning of one cue can vary considerably depending on the value of the other. Here we ask whether children show similar reciprocal influences in the perception of gaze and expression. A previous study has demonstrated that gaze direction affects the perception of emotional expression in children. Here we demonstrate the opposite direction of influence, showing that expression affects the perception of gaze direction. Specifically, we show that the cone of gaze, i.e., range of gaze deviations perceived as direct, is larger for angry than neutral or fearful faces in 8 year-old children. Therefore, we conclude that children, like adults, show reciprocal influences in the perception of gaze and expression. An unexpected finding was that, compared with adults, children showed larger effects of expression on gaze perception. This finding raises the possibility that it is the ability to process cues independently, rather than sensitivity to combinations, that matures during development. Alternatively, children may be particularly sensitive to anger in adult faces.

## Introduction

Sensitivity to combinations of expression and gaze cues is important for social interactions and social referencing [1]. The meaning of a smile or an angry expression can differ dramatically depending on the person's gaze direction. Adults are sensitive to interactions between expression and gaze cues, showing influences of gaze direction on the perception of expression [2]–[6] and of expression on the perception of gaze [7], [8] and combined head-gaze direction [9]. These reciprocal influences suggest that adults combine information about expression and gaze direction during face perception.

Sensitivity to cue combinations is adaptive and may originate early in development. Infants show neural sensitivity to combinations of expression and gaze direction [10]–[12]. For example, 4 month-olds show significantly more attention (larger Nc, an ERP indicator of attention) to happy than fearful expressions, but only for faces with direct gaze [12]. In addition, 7 month-olds show enhanced attention to angry faces with direct gaze compared to those with averted gaze [10]. This early sensitivity to combinations of expression and gaze direction may be important for social referencing, whereby infants use affective responses of others towards objects in the environment to evaluate those objects. Consistent with this idea, 6-month-olds show more attention to fearful than neutral faces when gaze is directed towards an object, but not when gaze is directed towards empty space, and they show more attention to objects that were previously looked at by a fearful face than a neutral face [11].

These infant studies suggest that neural markers of attentional responses to faces are sensitive to combinations of gaze and expression cues, but do not directly assess perceptual interactions between those cues. The youngest age at which these have been investigated is late childhood (M = 12, range 9–14 years) [13]. Akechi and colleagues found that gaze direction affected the perception of expression in this age group. Moreover, anger and fear were discriminated more quickly when paired with gaze directions that signaled a congruent (anger with direct gaze and fear with averted gaze) rather than an incongruent (anger with averted gaze and fear with direct gaze) motivational tendency. These results are consistent with the shared signal hypothesis, which proposes that perception of expression and/or gaze direction will be enhanced when both signal a common behavioral tendency, such as approach (e.g., anger and direct gaze) or avoidance (e.g., fear and averted gaze) [2], [3]. They also tested cognitively-able children with autism, who showed no congruence effect, suggesting deficient or delayed sensitivity to cue combinations in this population. These results indicate that gaze direction affects expression perception in typically developing children. However, no studies have tested whether expression affects gaze perception in children, so it is not yet known whether reciprocal effects between perception of gaze and expression occur in children.

The aim of the current study was to determine whether children show adult-like effects of expression on gaze perception, as required to demonstrate reciprocal effects of expression and gaze processing. There are several reasons to think that such effects would be found. First, sensitivity to expression and gaze combinations is important for early social referencing. Second, children as young as five years of age show adult-like interactions between other face cues (identity and expression, and identity and facial speech) when asked to sort on one cue and ignore the other [14]. Finally, the holistic coding mechanisms needed for perceptual integration of face cues are present in early childhood [15]. Nevertheless, neither gaze nor expression processing is fully mature in children, and it may well be that sensitivity to combinations of these cues is late to mature.

We tested eight-year-old children because they show adult-like performance in recognizing the high intensity fearful, angry and happy expressions used in our task [16], and near-adult performance in discriminating gaze direction [17]. Therefore, any reduction of, or failure to find, expression effects on the perception of gaze could not be attributed to difficulty perceiving the expressions or discriminating gaze directions. We used a modified, child-friendly version of Ewbank et al's (2009) cone of gaze task to measure the range of gaze deviations that are perceived as direct (i.e., the cone of gaze), in faces showing neutral, fearful and angry expressions. In their adult study, Ewbank et al. found that expression affected the cone of gaze. Specifically, the cones of gaze were larger for angry than neutral and fearful faces, with no difference in the cones of gaze for neutral and fearful faces. For the reasons outlined above, we predicted that children would also show these cone of gaze effects.

## Methods

### Participants

Thirty-two eight-year-olds (*M* = 8:7 years, *SD* = 3 months, range 8:1–8:11; 16 male) were recruited from schools in the Perth metropolitan area. Guardians provided informed consent prior to testing. Thirty-six Introductory Psychology students (*M* = 20 years, *SD* = 5 years, range = 17–48; 13 male) from the University of Western Australia provided informed consent and participated for course credit.

### Stimuli

**Figure 1:**
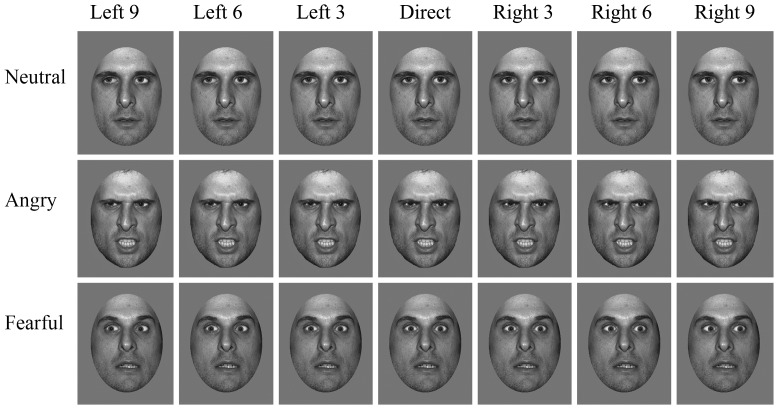
Neutral, angry and fearful expressions for three male identities showing the seven different eye gaze directions used here. This figure was adapted from Fig. 1 in [8] http://www.journalofvision.org/content/9/12/16.full?sid=62b18262-1084-4efe-8f2d-abb0c78eaf0f.

We used images of four male identities with angry, fearful and neutral facial expressions, taken from [8] (Figure 1). The faces had been rated on arousal and valence, and fearful and angry faces did not differ on either dimension [8]. For each expression, there were seven different gaze directions: 3, 6 and 9 pixels to the left, direct gaze, and 3, 6 and 9 pixels to the right (Figure 1). Two additional female identities with happy, angry, fearful and neutral expressions, were obtained from the NimStim Face Stimulus Set [18] for use in the practice trials. All faces were shown as grey-scale images, with hair, ears and neck masked. They subtended a visual angle of approximately 12°×8° when viewed from a distance of 50 cm.

### Procedure

All participants completed the cone of gaze task (25 mins) followed by an expression recognition task (five mins). These tasks were presented in the context of a game in which participants passed through six grades (four blocks of cone of gaze task plus 2 blocks of expression recognition task) before graduating from ‘Detective School’. Participants were asked to use their detective skills to look for the clues. The children received a sticker after each grade. The tasks were delivered using SuperLab Pro 4 Software on a lap-top with a 15-inch screen. Participants sat approximately 50 cm from the screen.

#### Cone of gaze task

This task was presented as the, ‘Where Am I Looking?’ game, in which they were a detective who had to indicate where the person on the screen was looking. It consisted of four blocks of 84 trials, with self-paced breaks provided after each block. Within each block, each identity (4), facial expression (3) and gaze deviation (7) combination was presented once. Trial order was randomized within each block for each participant. Participants pressed the space bar to initiate stimulus presentation for each trial, a face then appeared in the center of a grey screen for 400 ms. The task required participants to verbally indicate whether they perceived the face to be looking directly at them, left of them or right of them. Children who were confused by left and right, could point to where the face was looking. Responses were recorded on a keyboard by the experimenter.

Prior to the task, participants completed two blocks of practice trials using two female faces. Participants were told that the practice block was to make sure they were ‘ready for Detective School’. The first block showed these faces at each of three gaze directions: nine pixels left, nine pixels right and direct gaze. If an error was recorded, the trial was repeated until a correct response was recorded and feedback was then given. The second block was the same as the first but used five pixel deviations. Responses were recorded but erroneous trials were not repeated and no feedback was given. Trial order was randomized within each block.

#### Expression recognition task

The second task was presented as the, ‘What Am I Feeling?’ game, in which participants had to judge the expression of faces presented on the screen. The four male identities from the cone of gaze task, with neutral, fearful, angry and happy expressions, were shown. All had direct gaze. The task was split into two blocks. In the first block the faces were presented for 400 ms (as in the cone of gaze task). In the second block the faces were presented for unlimited exposure duration. Each block consisted of 32 trials, with each identity (4) and expression (4) combination presented twice. Participants had to verbally indicate whether they perceived the face to be angry, happy, fearful or neutral. These expressions were listed at the bottom of the screen when the response was prompted. Responses were recorded on a keyboard by the experimenter. Participants pressed the space bar to initiate each trial. Trial order was randomized.

Prior to the task, participants completed two practice blocks of four trials each. The first block had unlimited exposure duration, the second block had exposure duration of 400 ms (opposite order to the main task). Each expression was shown once in each block. No feedback was given. Trial order was randomized within each block.

## Results

### Cone of Gaze Task

Cone of gaze values were calculated for angry, neutral and fearful conditions for each participant (except one adult with very poor accuracy, 58%, for the most extreme gaze deviations). As in [8], separate logistic functions were fitted to the proportion of leftward and rightward responses. A function was then fitted to the proportion of direct responses, calculated by subtracting the sum of left and right fitted functions from one. These calculations resulted in nine functions overall, three for each expression. The cross-over points between the leftward and direct functions and rightward and direct functions, were calculated for each expression. The distance between these two crossover points was the cone of gaze for each expression. One child outlier whose angry cone of gaze was more than three standard deviations above the mean was excluded. A square root transformation was applied to the remaining data to reduce positive skew. The resulting distributions did not deviate significantly from normality.

**Figure 2:**
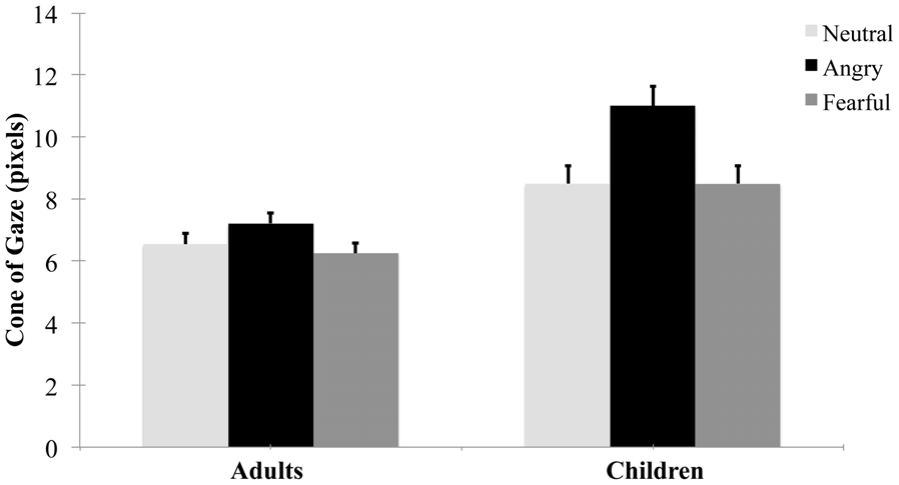
Untransformed cone of gaze values for each expression for children and adults. Error bars show ±1 SE.

The transformed cone of gaze values were entered as the dependent variable into a 3×2×2 mixed-model ANOVA, with expression as a within-participants factor (happy, neutral, angry) and age group (children, adults) and participant gender (male, female) as between participant factors. Gender was included for exploratory purposes as we had no predictions for gender. There was a significant main effect of expression, *F* (2, 124) = 43.28, *p*<.001, η^2^ = .41, with larger cones of gaze for angry (*M* = 2.95, *SD* = .54) than neutral (*M* = 2.68, *SD* = .52) or fearful (*M* = 2.66, *SE* = .47) faces (Figure 2). This effect was qualified by a significant interaction with age group, *F* (2, 124) = 9.03, *p*<.001, η^2^ = .13. Inspection of Figure 2 suggests that both children and adults had larger cones of gaze for angry faces than for neutral and fearful faces, as found previously for adults [8]. Planned comparisons conducted for each age group confirmed that this was the case. Adults showed significantly larger cones of gaze for angry faces than for neutral, *t*(34) = 3.74, *p* = .001, *d* = .36, or fearful, *t*(34) = 4.09, *p*<.001, *d* = .51, faces. There was no significant difference between the cone of gaze for neutral and fearful faces, *t*(34) = 1.06, *p* = .30, *d* = .11. Importantly, children also showed the adult pattern of significantly larger cones of gaze for angry than neutral, *t*(30) = 7.69, *p*<.001, *d* = .76, or fearful faces, *t*(30) = 6.71, *p*<.001, *d* = .79, and no significant difference between fearful and neutral faces, *t*(30) = .24, *p* = .81, *d* = −.02. These results indicate that children, like adults, show larger cones of gaze for angry than neutral or fearful faces.

Inspection of Figure 2, together with the interaction between expression and age group suggests that the influence of expression on gaze perception was actually larger for children than adults. To test this hypothesis, we divided the difference between angry and neutral cone of gaze scores by the neutral cone of gaze scores, to index the size of the increase relative to a neutral baseline. An independent samples t-test revealed that this increase was significantly larger for children (*M* = .35, *SD* = .27) than adults (*M* = .15, *SD* = .28), *t*(64) = 2.86, *p* = .006, *d* = .71, indicating a larger effect of expression on the cone of gaze in children than adults.

There was a significant main effect of age group, *F*(1,62) = 16.66, *p*<.001, η^2^ = .21, with larger cones of gaze for children (*M* = 3.00, *SE* = .08) than adults (*M* = 2.55, *SE* = .07). Given the significant interaction between age and expression, noted above, we examined whether this age effect was present for all three expressions. The cones of gaze were significantly larger for children than adults for all three expressions: angry faces, *t*(51.44) = 5.46 *p*<.001, *d* = 1.36, neutral faces, *t*(64) = 2.82, *p* = .006, *d* = .69, fearful faces, *t*(64) = 3.77, *p*<.001, *d* = .92. Levene's test for equality of variances was violated for the angry expressions, so a *t* statistic not assuming homogeneity of variance was conducted in that case.

There was no main effect of gender, *F* (1, 62) = .27, *p* = .60, η^2^ = .004, and no significant interaction of gender with either expression, *F* (2, 124) = 1.89 *p* = .15, η^2^ = .03, or age group, *F* (2, 124) = 1.79, *p* = .19, η^2^ = .03.

### Expression Recognition Task

As expected, adults recognized the expressions almost perfectly in both the 400 ms (*M* = 97.2, *SD* = 7.3) and unlimited exposure (*M* = 97.6, *SD* = 6.7) conditions. Children also performed extremely well in both conditions (*M* = 95.0, *SD* = 11.1, 400 ms; *M* = 96.2, *SD* = 11.4, unlimited).

## Discussion

We found that eight-year-old children, like adults, perceive a wider range of gaze directions as direct when faces display angry, compared with neutral or fearful, expressions. Therefore, expression affects gaze perception in children as well as in adults. The opposite effect, of gaze direction affecting expression perception, has also been found in slightly older children (M = 12 years, range 9–14) [13]. Taken together these two findings demonstrate that reciprocal processing of expression and gaze is in place by late childhood. More generally, they show that children are sensitive to combinations of gaze and expression cues.

Although the children in our study showed sensitivity to expression when judging gaze direction, their performance was not completely adult-like. First, they had larger cones of gaze for neutral faces than did adults. This result is consistent with other evidence that the cone of gaze is wider in 8 year-olds than adults [17]. Although Vida and Maurer (2012) found wider cones of gaze for 8 year-olds than adults, this difference was not statistically significant, leading them to conclude that perception of direct gaze is adult-like by 8 years of age. However, the present results suggest that their result may be due to lower power due to a smaller sample (N = 18 in each age group, cf. 35 in the present study). Our results suggest that sensitivity to gaze direction may continue to develop throughout childhood, along with other aspects of face expertise [16], [19]–[28].

The second way in which children's performance was not adult-like was more surprising. Their perception of gaze direction was *more* influenced by expression than that of adults. Specifically, angry expressions increased children's cones of gaze more than adults' cones of gaze, even controlling for their larger cones for neutral faces. This result raises the intriguing possibility that it is the ability to process cues independently that matures during development, rather than the ability to integrate cues. If this conjecture is correct, then children's perceptions of expression should also be more affected by gaze direction than those of adults. This prediction remains to be tested. Several factors could contribute to increasing independence of gaze and expression processing during development. An obvious candidate is improvements in top-down control of visual selective attention [29]. Another, more speculative possibility, suggested by individual differences in adults, is that faster processing of cues results in less interaction [1], [7].

Tasks that require participants to sort faces according to one cue (e.g., expression) and ignore another (e.g., identity) provide mixed support for our conjecture that processing of face cues may become more, rather than less, independent during development [14], [30]. Chapman (1981) found integral processing of eyes (open or closed) and mouth (smiling or frowning) in 6 year-olds but not 9 year-olds, suggesting an increase in independence. In contrast, Spangler et al. (2012) found no developmental change in interference from identity variation when sorting by expression and facial speech (across the ages of 5–11 years and adults). However, no studies have yet examined interactions between expression and gaze in a sorting task, and it may be useful to do so in the future. The present results suggest that expression should interfere with sorting faces by gaze direction and that the interference should be larger for children than adults.

An alternative interpretation of the larger influence of angry expressions on childrens' than adults' cones of gaze, found in the present study, is that angry adult male faces provide more salient signals of threat or disapproval, and therefore larger influences on gaze perception, for children than adults. A related possibility is that children may experience adult anger directed at them more frequently than adults do, which could lower their criterion for interpreting such expressions as being directed towards them.

Ewbank et al (2009) have shown that the larger cone of gaze for angry faces in adults is not due simply to difficulty seeing the position of the iris in angry faces, which have narrowed eyes. If it was, then the same effect of expression should be found for inverted faces, even though expressions are difficult to perceive in such faces. However, this was not the case. For inverted faces, the cone of gaze was virtually identical for angry, neutral and fearful faces [8]. We did not include inverted faces in the present study, so that we could keep testing to a single session for children. However, many studies find that visual acuity is mature by 8 years of age [31], suggesting that low-level visibility effects are unlikely to differ for children and adults.

An influential account of reciprocal effects of gaze and expression perception is the shared signal hypothesis, which proposes that perception of cues is enhanced when they signal the same behavioral or motivational tendencies, either approach (e.g., anger and direct gaze) or avoidance (e.g., fear and averted gaze) [2], [3]. According to this hypothesis there should be a larger cone of gaze for angry than neutral expressions and a smaller cone of gaze for fearful than neutral expressions. Although we found a larger cone of gaze for angry than neutral faces, we found no evidence for a reduced cone of gaze for fearful faces, either in adults or in children. Our adult results replicate previous findings [8]. In addition, several studies have also failed to find the opposite effect, of averted gaze facilitating the perception of fear [4], [32]–[34]. The shared signal hypothesis does not, therefore, seem to account for the full range of findings (unless both neutral and fearful expressions are avoidant signals and are perceived categorically rather than continuously). As an alternative account of the cone of gaze results, we suggest that larger cones of gaze for angry than neutral or fearful faces may result from an adaptive bias to err on the side of false positives when deciding whether or not anger is directed towards oneself. Our results suggest that this bias may be particularly strong in children, at least when confronted by adult male faces displaying anger.

The present study is the first to demonstrate that expression affects the perception of gaze direction in children. Given that gaze direction affects the perception of expression in children [13], we conclude that children show reciprocal influences between gaze and expression cues when perceiving faces. These influences may play an important role in social perception because the meaning of one cue can depend on the value of another. For example, an angry face has very different meanings depending on whether its gaze is direct or averted. Surprisingly, children showed larger effects of expression on gaze perception than did adults. This result could reflect a stronger adaptive bias to interpret anger as directed at the self. Alternatively it could reflect greater difficulty in selectively attending to one cue (gaze direction) and ignoring others (expression). Future studies are needed to determine how the reciprocal processing of gaze and expression develops during childhood and adolescence, and the contributions from development of face-selective and more general selective-attention mechanisms.
